# Bristol’s and Edward’s Shoes

**Published:** 1877-12

**Authors:** 


McComber's Shoes.—It would be a safe estimate to say that one-half the adult population of the country have corns, bunions, or other deformities of the feet. These evils have been brought on by wearing shoes illy adapted to the shape and size of the feet.

These injuries to the feet, beside the pain and deformity they cause, are productive of permanent injury to the general health, by interrupting the circulation of the blood in the lower extremities. W. Bristol & Co., No. 8 Warren Street, New York, are manufacturers of ladies' and children's shoes. We have never seen shoes that equalled theirs, in fit, beauty, or durability. This firm has the exclusive right to manufacture ladies' shoes in New York on McComber's patent lasts.

Mr. Bristol says that any shoe that requires *breaking* as it is called, is unfit to be worn. He thinks shoes should be made to fit the feet, and that the feet should not be compelled to fit the shoes, as is almost always the case. Those who desire to experience the luxury of wearing a beautiful pair of glove fitting shoes without undergoing the torture of breaking them before they can be worn with comfort, and who can not find McComber's shoes at retail stores, should call at Bristol & Co's shoe manufactory, and select their shoes or leave their measure.

Since writing the above it has occurred to us that in our desire to render a service to the "fair sex" we were near forgetting that men also are great sufferers through the ignorance of our modern Crispins. About four years ago we purchased a pair of shoes, made on the McComber patent last, by F. Edwards of 166 and 168 Atlantic Avenue, Brooklyn. We put them on and wore them without inconvenience from the first day. Since then we always wear shoes and boots made on this plan, and do not intend to wear any other.

